# New Homes

**Published:** 1874-03

**Authors:** 


### NEW HOMES.

### CALIFORNIA.

BETWEEN the professional scribe who visits a country to "write it up" and the disappointed man who leaves it to write it down, it is difficult to get at the facts. From the mass of material at our disposal we have selected a letter published in the *Cincinnati Herald and Presbyter* from its correspondent in San Jose, Cal., which seems so free from prejudice in either direction that we print most of it.

In reference to the Santa Clara Valley, after seven months' residence there, he writes :

" A great deal more depends upon the immigrant, his energy, his grit, his power of adapting himself to circumstances, his habits of industry and economy, than upon the soil or climate of the region to which he goes. There are men here who came early, who have worked hard, men of good habits and talents, who are not worth a hundred dollars. They rent land—the owner furnishing the teams and tools and getting half of the crops, or they have teams, and pay in money for the use of land.

" I asked one of these men to-day: 'Why don't you buy a small farm?' His answer was, 'How could I ever pay for it? The land that I rent for $6 an acre I could not buy for less than $125 an acre. On all the deferred payments I should have to pay 12 per cent. per annum. Hence, the yearly interest would be $15 an acre while the rent is only $6.' 'Well, how much can you raise on an acre of this land?' 'In good seasons three tons of wheat or barley hay to the acre, and this hay is worth usually about $10 a ton after harvest, but it costs about $3 a ton to get it baled. Hence, the net value of the crop is about $24 to the acre, and the renter gets, in a good year, about $18 to the acre for his labor. But occasionally there is a dry year, when he will not get more than enough to pay his rent.'

"But suppose a man has $2,000 or $3,000 to start with. Well, it will cost a good deal to get here—about $150 for each person and $6 to $9 for each hundred pounds of household goods or furniture. Then, on getting here, the greenbacks must be turned into gold at a discount of 10 or 11 per cent. A man's $3,000 would be reduced to $2,000 in gold before he was ready to buy his farm. He could get with that from twenty to fifty acres of valley land, without any building, or from fifty to one hundred acres of hill land (half of it too steep for anything but pasturage). Now, if the immigrant was willing to work hard and live close for a few years, until he could get a portion of his land covered with choice fruit trees in full bearing, he might secure quite a good income; but if he falls behind and has to borrow, he is ruined. No man can pay 12 or 15 per cent. per annum on land for farming purposes.

"There is a great deal of land in California that can be bought at Government prices, or less. But from all I can learn about it I think it not so desirable as the cheap lands in Kansas and Nebraska. Here all the valuable land accessible to market, or likely to be, was taken up years ago. Speculators and corporations own immense tracts, and don't mean to sell them without a profit.

"We have a very large State, but a great portion of it is filled with granite mountains. Immense tracts are sandy and arid. Other tracts, like the San Joaquin (pronounced San Wakeen) Valley, are very productive in seasons when there is a great deal of rain, but the crops fail entirely—literally burn up—one third of the time (some say two thirds). Other tracts, called Tule lands, are marshy and malarious. Nearly all of the really good land is in a few valleys, like Sacramento, San Rafael and Santa Clara, and in these valleys, as a rule, it is in a few hands and held at high figures.

"Sheep raising here thus far has been conducted only on a large scale. A man who has less than 10,000 is hardly considered in the business at all. These immense flocks are pastured on Government land in remote sections. The pasture costs nothing, and, of course, the profits are large. Yet few persons would care to live in the wilderness even for such profits; and the wilderness itself is being so circumscribed by the progress of civilization that these vast flocks, or bands, as they call them, will soon have to be broken up. The sheep business is in a transitive state, and the question now is, Will sheep pay on pasture land that costs $10 to $20 an acre? or will it pay to feed sheep on alfalfa, where land is worth from $50 to $100 an acre? The climate is favorable. A band of sheep will double every year; but the wool, on account of the warm winters, is of inferior quality. It brings in market only about half as much as eastern grown wool. They shear sheep here twice a year—in the spring and in the fall. The spring clip is the best. It brings from five to ten cents a pound more than the fall clip. My impression is that a fair business can be done in sheep farming on a small scale, in bands of from one to five hundred. Five hundred sheep well cared for would yield 3,000 pounds of wool, worth now about $600. The five hundred lambs would be worth, when a year old, $1,000, making the gross proceeds $1,600. The amount of land required to keep this band of sheep would depend on its quality and the season. But say 200 acres of hill land at $20 an acre, $4,000; cost of sheep, $1,000 to $1,500; sheepfolds, cottage for shepherd, etc., $500, whole cost, $6,000; board and wages of shepherd, $600 a year; interest on investment, $600; net profit, $400. Out of this $400 would have to cover the contingent expenses and the losses incident to all kinds of business. But it looks as if there might be 'the probability for a conjecture' that sheep will pay. Yet it is only conjectured, for sheep farming on a small scale and on a limited range has not been tried here yet to any extent.

"Fruit growing is a good business here for those who can afford to buy land; then buy trees and plant them with care, and then wait until the trees come into bearing. Orchards of choice varieties in favorable years will bring in from $50 to $100 an acre, but the cost of that acre and the culture of it for four or five years is a pretty heavy investment. No man can get a fruit farm large enough to support a family without spending on it at least $4,000 to $5,000, and he will get very little income from the land until his trees begin to bear.

" Grain growing in California is conducted on a large scale by wealthy landowners who use a great deal of machinery. A Mr. Glenn, of Colusa, is putting in this year 40,000 acres of wheat. His estimated crop is 1,000,000 bushels, or 30,000 tons, enough to load twenty ships of 1,500 tons burden. Men with small farms and little capital to invest in machinery cannot compete with such farmers. If there comes a dry season Mr. Glenn lives on his accumulations of former years, discharges his hands and lets his land rest. If the price of wheat is low he stores it until there is an advance. He can afford to farm in such a region of variable rain fall as this, but the small farmer, who has nothing but his land, is starved when a dry year comes.

" Farmers here usually raise only one kind of crop. The wheat grower plants nothing but wheat. He buys all his potatoes, turnips, cabbages, etc.; also his fruit. I see farmers at the fruit and vegetable stalls in San Jose buying what every farmer in Ohio raises. One reason is that almost everything but grain requires irrigation. The vegetable garden must be watered, and this is too much trouble for the man who is cultivating thousands of acres with grub ploughs, headers, steam threshers, etc.

" Many here are beginning to think and say that the present system of farming is unfavorable to the best interests of the country, that it would be much better to have the land cut up into smaller farms, and to have each farmer raise a greater variety of crops. Water can be obtained in all our valleys by digging. Many of the wells are artesian, and the flow of water from one of them will irrigate from twenty to fifty acres. Where the water does not flow it is easily pumped by wind mills. The winds here are so steady that the mills work admirably. But these wells and mills cost a great deal of money. I think that a good farmer who should buy here a farm of moderate size and cultivate it as farms are cultivated in 'the States,' would do better in the long run than those California farmers with their one idea style of doing things.

" Then, to those who have 'the California fever,' I would say, What has given you that fever? Is it a desire to improve your worldly circumstances merely? If so, you will see from my statements that I do not consider this a particularly eligible place for you to come to. I believe that more money can be made with the same capital, toil and skill in other localities. The time for big speculations, for picking up nuggets of gold, for getting $8 a day for mechanical labor has gone by. California is well supplied with all kinds of skilled laborers, with professional men, with teachers, with shopkeepers, with clerks, with agents and operators. There are more men wanting to rent land than there is land to be rented. There are more men wanting to herd sheep than there are bands of sheep to be herded. There is close competition, and a supply fully equal to the demand in every department of industry and enterprise. I know persons who have been here for months seeking remunerative employment without success.

"But if severe winters and failing health have caused 'the fever;' if you cannot enjoy life where you are; if you feel that you must get into a milder climate at any sacrifice, then my advice is: Come to this valley or some similar valley on this coast. Come, expecting to labor and economize. You will, no doubt, be able to get a living, and you will greatly enjoy the balmy air, the fine scenery, the new varieties of tree and flower, and the luscious fruits. It is a splendid country for those who want to be as much as possible in the open air."

